# The Role of HDACs and HDACi in Cartilage and Osteoarthritis

**DOI:** 10.3389/fcell.2020.560117

**Published:** 2020-09-30

**Authors:** He Zhang, Lu Ji, Yue Yang, Xiaoning Zhang, Yi Gang, Lunhao Bai

**Affiliations:** ^1^Department of Orthopedic Surgery, Shengjing Hospital, China Medical University, Shenyang, China; ^2^Department of Gynecology and Obstetrics, Shengjing Hospital, China Medical University, Shenyang, China; ^3^Department of Anesthesiology, Shengjing Hospital, China Medical University, Shenyang, China; ^4^Department of Orthopedic Surgery, Panjin Central Hospital, Panjin, China

**Keywords:** osteoarthritis, HDAC, HDAC inhibitor, microRNA, cartilage

## Abstract

Epigenetics plays an important role in the pathogenesis and treatment of osteoarthritis (OA). In recent decades, HDAC family members have been associated with OA. This paper aims to describe the different role of HDACs in the pathogenesis of OA through interaction with microRNAs and the regulation of relevant signaling pathways. We found that HDACs are involved in cartilage and chondrocyte development but also play a crucial role in OA. However, the distinct HDAC mechanism in the pathogenesis and treatment of OA require further investigation. Furthermore, HDAC inhibitors (HDACi) can protect cartilage from disease, which may represent a potential therapeutic approach against OA.

## Introduction

Osteoarthritis (OA) is a common disease that not only causes physical disability but also imposes an economic burden on society (Litwic et al., 2013; Liu et al., 2018). The prevalence of OA is high and increases with age (Neogi, 2013). In Korea, 35% of people older than 65 years have been diagnosed radiographically with OA (Cho et al., 2015). The etiology of OA is multifactorial and complex. Mechanical stress, metabolic dysfunction and inflammation are all involved in OA progression (Sarzi-Puttini et al., 2005; Johnson and Hunter, 2014). Due to the aging population and the rising rate of obesity, the prevalence of OA is predicted to double by 2020 (Thomas et al., 2017).

OA is characterized by joint space narrowing, subchondral sclerosis, subchondral cysts, and osteophyte formation (Kuyinu et al., 2016). Its major clinical symptoms include joint pain and swelling and loss of movement (Shen and Chen, 2014; Moon et al., 2018). The pathological mechanism of OA includes increased dysfunction and death of chondrocytes and the disequilibrium of extracellular matrix synthesis and degradation (Zheng et al., 2018). There are many signaling pathways involved inOA pathogenesis that are activated by pro-inflammatory mediators and cytokines, such as interleukin-1β (IL-1β) (Jenei-Lanzl et al., 2019). Specifically, these cytokines promote OA through mitogen-activated protein kinase (MAPK) signaling (Malemud, 2017), NF-κB, and other signaling pathways (Rigoglou and Papavassiliou, 2013; Jenei-Lanzl et al., 2019). The activation of catabolic signaling pathways and inhibition of anabolic signaling pathways lead to overexpression of matrix metalloproteinases (MMPs) and a disintegrin and metalloproteinase with thrombospondin motifs (ADAMTS).

Treatments for OA are developing rapidly. Platelet-rich plasma (PRP), mesenchymal stem cells (MSCs) and physical therapy are extensively applied to treatment of OA (Bennell et al., 2014; Bennell et al., 2017; Si et al., 2017; Toh et al., 2017). However, current medical management only focuses on the relief of symptoms, not the reversal of OA progression. It’s unavoidable that OA patients suffer from the side effects of treatment. Therefore, it’s essential to identify new therapeutic interventions for OA.

Recently, multiple studies have demonstrated that altered activity, expression, and distribution of histone deacetylases (HDACs) lead to the initiation and progression of OA. HDAC inhibitors (HDACi) can protect chondrocytes and prevent cartilage damage (Khan and Haqqi, 2018). This review focuses on the following insights: (1) the relationship between each HDAC and OA; (2) the relevant mechanisms governing HDACs involvement in OA; (3) the potential of HDACi in OA treatment.

## HDAC Structure and Function

**FIGURE 1:**
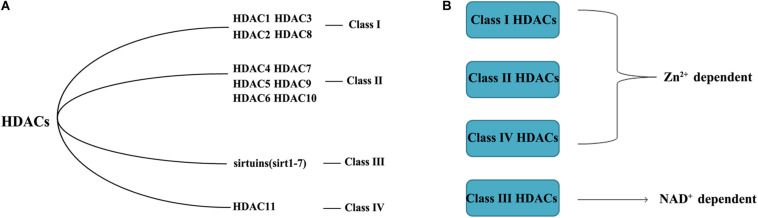
Classification of HDACs **(A)** and the different mechanism of action **(B)**.

HDACs, also called lysine deacetylases, are nuclear transcriptional regulatory proteins that regulate chromosome structure and the activity of transcription factors by removing acetyl groups from histones (Araki and Mimura, 2017). HDACs and histone acetyltransferases (HATs) are the two major components that maintain a balance in transcription activity, with HDACs inhibiting gene activation (Kroesen et al., 2014). The substrates of HDACs are abundant; HDACs can modify more than 3600 acetyl groups of over 1750 proteins. There are currently 18 HDACs, divided into four groups (Figure 1): Class I(HDAC1, HDAC2, HDAC3, and HDAC8), Class II(HDAC4, HDAC5, HDAC6, HDAC7, HDAC9, and HDAC10), Class III (sirtuins, sirt1–7), and Class IV (HDAC11). Class I and II HDACs require Zn^2+^ to maintain enzyme activity. The Class III HDACs are NAD^+^ -dependent (Hesham et al., 2018), and Class IV consists of a single HDAC11 (de Ruijter et al., 2003). Class I HDACs exist mostly in the nucleus, expect for HDAC3 and HDAC8, which can shuttle between the nucleus and cytoplasm (Hull et al., 2016). The distribution of Class I HDACs is highly tissue-specific (Yoon and Eom, 2016). Most Class II HDACs are located in both the nucleus and cytoplasm and need to recruit Class I HDACs to obtain catalytic activity (Carpio and Westendorf, 2016).

In addition to transcriptional regulation, HDACs are involved in posttranslational modifications (PTMs). PTMs determine protein activity, stability, distribution and interaction (Yoon and Eom, 2016). Non-histone proteins such as NF-κB, heat shock protein (HSP), P53, signal transducers and activators of transcription (STAT), forkhead transcription factor (FOXO) and mitogen-activated protein kinase (MAPK) are all modified by HDACs to regulate biological pathways (Gallinari et al., 2007; Spange et al., 2009; Jeong et al., 2014; Leus et al., 2016). Previous studies focused on the role of HDACs in cancer, pulmonary fibrosis, cardiovascular disease and rheumatoid arthritis (Ooi et al., 2015; Angiolilli et al., 2017; Lyu et al., 2019). However, there are few articles indicating a role for HDACs in OA.

Class III HDACs (sirtuins) differ from the Class I and II HDACs structurally and mechanistically (NAD^+^- dependent) (Dvir-Ginzberg et al., 2016). Meanwhile, there have been no reports indicating a role for the Class IV HDAC in OA. Therefore, we primarily discuss the role of the Class I and Class II HDACs in cartilage development and OA progression and the potential therapeutic effects of Class I and Class II HDAC inhibitors.

## HDACs and miRNA in OA

MicroRNAs (miRNAs) are non-coding RNAs that regulate gene expression through post-transcriptional modifications. Altered miRNA expression is found in many diseases, including OA (Sondag and Haqqi, 2016). There have been many studies that identified a relationship between HDACs and miRNA in pathogenesis of OA (Table 1).

**TABLE 1:** The relationship between HDACs and miRNAs in OA.

**HDAC**	**miRNA**	**Differential expression in OA**	**Target gene(s)**	**miRNA Biological effects**	**References**
HDAC2	miR-95-5p	Downregulated	HDAC2/8	Matrix synthesis	Mao et al. (2018)
	miR-92a-3p	Downregulated	HDAC2	Matrix synthesis	Mao et al. (2018)
	miR-455-3p	Downregulated	HDAC2/8	Matrix synthesis	Chen et al. (2016b)
HDAC3	miR-193b-3p	Downregulated	HDAC3	Matrix synthesis	Meng et al. (2018)
HDAC4	MiR-381	Upregulated	HDAC4	Matrix degradation	Chen et al. (2016c)
	miR-438-5p	Upregulated	Matn3 and TIMP2	Chondrocyte hypertrophy and angiogenesis	Wang et al. (2018a)
	miR-365	Upregulated	HDAC4	Inhibiton of MMP13 and Col X gene expression	Yang et al. (2016)
	miR-222	Downregulated	HDAC4	Inhibition of chondrocyte apoptosis and MMP13	Song et al. (2015)
HDAC7	miR-193b-5p	Downregulated	HDAC7	Matrix synthesis	Zhang et al. (2019a)
HDAC8	miR-95-5p	Downregulated	HDAC2/8	Matrix synthesis	Mao et al. (2018)
	miR-455-3p	Downregulated	HDAC2/8	Matrix synthesis	Chen et al. (2016b)

## HDAC1

MiR-146a has a protective effect against OA by inhibiting inflammatory factors in cartilage and synovial tissues (Yang et al., 2014; Guan et al., 2018). In contrast, HDAC1 inhibits miR-146a expression in the synovium to aggravate cartilage damage (Wang et al., 2013). It’s unclear whether HDAC1 regulates miR-146a expression in cartilage in OA.

## HDAC2

HDAC2 has a similar structure to HDAC1 and also acts as a pro-inflammatory protein in OA pathogenesis (Brunmeir et al., 2009). Increased HDAC2 expression is observed in the cartilage and chondrocyte-secreted exosomes of OA patients and inhibits cartilage-specific gene expression in chondrocytes (Hong et al., 2009). Exosomal miR-95-5p delays OA progression and promotes cartilage matrix expression in chondrocytes by binding to the 3′-UTR of HDAC2 and inhibiting HDAC2 expression (Mao et al., 2018). Similarly, miR-92a-3p promotes cartilage matrix gene expression both in chondrogenic hMSCs and primary human chondrocytes (PHCs) by inhibiting HDAC2 expression through binding to the 3′-UTR of HDAC2 followed by increased H3 acetylation on the Aggrecan (ACAN), COMP and Col2a1 promotors and increased cartilage matrix expression (Mao et al., 2017). MiR-455-3p also has a protective effect on cartilage by inhibiting to the 3′-UTR of HDAC2, decreasing its expression, and promoting H3 acetylation on the Col2a1 promoter (Chen et al., 2016b).

## HDAC3

HDAC3 is involved in the repression of cartilage matrix metabolism (Zhang et al., 2019b). MiR-193b-3p targets the 3′-UTR of HDAC3 and inhibits its expression. HDAC3 suppression results in H3 acetylation and over-expression of Col2a1, ACAN, COMP, and SOX9 in hMSCs and PHC with or without IL-1β stimulation (Meng et al., 2018).

## HDAC4

The role of HDAC4 in OA has not been defined. Mammalian target of rapamycin complex 1 (mTORc1) activation induces extra cellular matrix (ECM) degradation through miR-483-5p-mediated downregulation of Matn3 and tissue inhibitor of metalloproteinase-2 (Timp2) in a Col2a1TSC1KO OA mouse model (Wang et al., 2018a). HDAC4 reverses OA symptoms by inhibiting miR-483-5p (Wang et al., 2018a). MiR-381 aggravates cartilage degradation and OA progression, whereas HDAC4 reverses this effect. The underlying mechanism involves the inhibition of HDAC4 expression by miR-381 binding to its 3′-UTR. Such inhibition decreases MMP13 and Runt-related transcription factor 2 (Runx2) expression in ATDC5 chondrocyte and SW1353 chondrosarcoma cell lines (Chen et al., 2016c). MiR-365 also promotes osteoarthritic cartilage destruction by targeting HDAC4 (Yang et al., 2016). Interestingly, HDAC4 also acting as a pro-inflammatory factor, can accelerate OA progression by inhibiting miR-146a in osteoarthritis synovial fibroblast-like cells (OA-FLS) (Wang et al., 2013). This contradictory phenomenon may be explained by the fact that this latter study only focused on the effect of HDAC4 on miR-146a and the downstream proteins interleukin-1 receptor-associated kinase 1 (IRAK1) and tumor necrosis factor receptor-associated factor 6 (TRAF6), but HDAC4 can be a positive regulator in other processes. Thus, overall, HDAC4 has anti-inflammatory and anti-arthritis effect. The second reason might be that HDAC4 acts as a pro-inflammatory factor in FLS but not chondrocytes. However, Song et al. (2015) found that miR-222 over-expression suppressed chondrocyte apoptosis and MMP13 expression by inhibiting HDAC4. Thus, the distinct mechanism of HDAC4 in OA requires further investigation.

## HDAC5 and HDAC6

There are no article about relationship between HDAC9, HDAC10, and miRNAs in pathogenesis of OA.

## HDAC7

HDAC7 evokes cartilage damage and ECM degradation through the over-expression of MMP3 and MMP13. In contrast, miR-193b-5p protects cartilage from injury by inhibiting HDAC7 through binding to its 3′-UTR (Zhang et al., 2019a). Interestingly, HDAC7 inhibition by siHDAC7 also promotes miR-193b-5p expression, suggesting that HDAC7 regulates miR-193b-5p in OA via a positive-feedback loop.

## HDAC8

Like HDAC2, HDAC8 exacerbates OA by inhibiting matrix metabolism, whereas miR-455-3p and miR-95-5p suppress HDAC8 expression to protect cartilage as mentioned above (Chen et al., 2016b; Mao et al., 2018).

## HDAC9 and HDAC10

There are no article about relationship between HDAC9, HDAC10, and miRNAs in pathogenesis of OA.

## HDACs and Signaling Pathways in Cartilage Development and OA

### HDAC1

HDAC1 expression is elevated in OA cartilage (Hong et al., 2009). The carboxyl-terminal domain (CTD) of HDAC1 is the major regulatory unit in OA pathogenesis. While the CTD does not determine HDAC1 enzymatic activity, it does affect the target gene specificity. The HDAC1 CTD promotes Snail1 transcription factor activity, a known repressor of Collagen2 (α1) in chondrocytes (Hong et al., 2009). Leukemia/lymphoma-related factor (LRF) suppresses expression of the COMP gene. HDAC1 increases LRF activity and suppresses COMP transcription in chondrocytes (Liu et al., 2004). In addition, HDAC1 assists HDAC9 in weakening Nkx3.2 stability by regulating acetylation status, which is required for chondrocyte viability and chondrocyte hypertrophy (Choi et al., 2016). Interestingly, there have also been controversial observations. During chondrocyte proliferation and chondrogenesis, zinc finger nuclear regulator (Trps1) plays a critical role in mitosis. An interaction between Trps1 and HDAC1 increases the histone deacetylase activity of HDAC1, leading to normal chondrocyte mitosis (Wuelling et al., 2013). HDAC1 also promotes cartilage development through the canonical Wnt/β-catenin pathway. HDAC1 suppresses β-catenin expression through its promoter and increases β-catenin degradation by regulating acetylation (Huang et al., 2014). Thus, HDAC1 plays a positive role in early cartilage formation and development but has a negative role in OA pathogenesis.

### HDAC2

The HDAC2 CTD also interacts with the Snail transcription factor to promote its activity and inhibit COMP expression (Hong et al., 2009). Protein kinase epsilon (PKCε) increases SOX9 expression, the deposition of glycosaminoglycans (GAGs) and inhibition of Runx2 expression in OA through HDAC2 down regulation (Queirolo et al., 2016).

### HDAC3

HDAC3 plays an important role in the development of bone and cartilage but can also exacerbate OA progression. HDAC3 is required for chondrocyte maturation at the early stage of skeletal formation in mice (E10.5 and E16.5). Postnatal ablation of HDAC3 in chondrocytes delays chondrocyte endochondral maturation, ossification and induces inflammatory cytokines in normal chondrocytes (Carpio et al., 2016). HDAC3 also inhibits the Erk1/2 downstream proteins (Runx2 and MMP13) and promotes chondrocyte maturation in the growth plate, which inhibits temporal and spatial activation of Erk1/2 through the up-regulation of the dual-specific phosphatase Dusp6 (Carpio et al., 2017). HDAC3 also represses Phlpp1 transcription to promote Akt phosphorylation and activation of its downstream targets (mTOR and p70 SK6) in chondrocytes. These events are essential for regulating chondrocyte hypertrophy and the promotion of matrix gene expression (Bradley et al., 2013). In the pathogenesis of OA, HDAC3 promotes OA progression via the regulation of the nuclear transportation of NF-κB in OA cartilage and chondrocytes with elevated MMP13 and ADAMTS5 expression (Zhang et al., 2019b). The contradictory phenomenon may be explained by the following: (1) HDAC3 is only essential for chondrocytes during the embryonic growth period; (2) HDAC3-deletion may slightly elevate inflammatory cytokines compared to normal chondrocytes, but significantly inhibit inflammation compared to chondrocytes treated with IL-1β.

### HDAC4

HDAC4 is the most thoroughly studied HDAC in OA pathogenesis. Decreased HDAC4 expression is observed in OA patients. HDAC4 not only inhibits the expression of Runx2, MMP1, MMP3, MMP13, ADAMTS4, and ADAMTS5 but also partially blocks the catabolic events in chondrocytes stimulated by IL-1β (Cao et al., 2014). As mentioned earlier, PKCε promotes SOX9 expression and the deposition of GAGs in chondrocytes via HDAC4 up-regulation (Queirolo et al., 2016). The PTHrp-Zfp521-HDAC4 pathway could negatively regulate chondrocyte hypertrophy. Zfp521 is a downstream target gene of PTHrp and forms a complex with HDAC4 and Runx2, leading to the repression of Runx2-mediated target gene activation (Correa et al., 2010).

Alterations in HDAC4 and cellular localization can regulate chondrocyte hypertrophy, OA progression and affect chondrocyte hemostasis. PTHrp promotes the nuclear translocation of HDAC4 and inhibition of MEF2 transcriptional activity to prevent chondrocyte hypertrophy (Kozhemyakina et al., 2009). In addition, HDAC4 is a mechanical-responsive protein; its expression can be regulated by mechanical compression in chondrocytes. Hydrostatic pressure (1–5 Hz) significantly decreases HDAC4 expression in OA chondrocytes to maintain the chondrocyte phenotype (Cheleschi et al., 2017). Furthermore, mechanical stimulation also alters the subcellular distribution of HDAC4 in these cells. Proper compression of chondrocytes promotes matrix-related gene expression through HDAC4 translocation to the nucleus (Chen et al., 2016a). This effect is dependent on PP2A-induced HDAC4 dephosphorylation. The relocation of HDAC4 associated with 14-3-3 to the cytoplasm also promotes CaMK IV-induced expression of Runx2 and related proteins in the chondrocytes (Guan et al., 2012). Interestingly, HDAC4 also has a destructive role in OA. HDAC4 is an upstream mediator of MAPK and promotes ADAMTS4, ADAMTS5, and cyclooxygenase 2 (Cox2) expression in rat articular chondrocytes stimulated with IL-1β (Wang et al., 2018b). The reason for the inconsistent phenomenon is unclear and still need further explored.

### HDAC5

Little is known on the role of HDAC5 in cartilage development and OA progression. HDAC5 acts as a co-activator of HDAC4 to inhibit chondrocyte hypertrophy through parathyroid hormone-related protein (PTHrP), which blocks the Mef2/Runx2 signaling pathway (Cheng et al., 2019).

### HDAC6

Mechanical intervention and physical activity can modify the epigenetic state by regulating the HDACs. This process is called mechanic-epigenetics (Chen et al., 2013). HDAC6 is a mechanosensitive protein involved in OA. It promotesADATMS5 expression through cilia disassembly and hedgehog signaling at 0.33 Hz and 20% cyclic tensile strain (CTS). This effect is not observed at 10% CTS (Thompson et al., 2014). Mechanical loading also attenuates NF-κB activity in chondrocytes stimulated with IL-1β through the regulation of the intraflagellar transport (IFT)-dependent pathway. Under the conditions of 0.33 Hz and 10% CTS, HDAC6 activity increases followed by the recovery of cartilage (Fu et al., 2019).

### HDAC7

HDAC7 plays a pivotal role in both cartilage development and OA. Increased HDAC7 expression is observed in the knee cartilage of OA patients, and HDAC7 induces MMP13 overexpression in OA (Higashiyama et al., 2010). Furthermore, insulin-like growth factor 1 (Igf1)/insulin-dependent signaling activates β-Catenin signaling, which promotes chondrocyte proliferation in immature chondrocytes. Igf1/insulin pathway also promotes HDAC7 translocation from the nucleus to the cytosol, where it is degraded by the proteasome (Bradley et al., 2015).

### HDAC8

HDAC8 promotes JNK phosphorylation to increase the expression of ADAMTS4, ADAMTS5, ColX, and Cox-2 in chondrocytes (Wang et al., 2018b). This effect is inhibited by both HDAC8 siRNA and the HDAC8 inhibitor, PCI.

### HDAC9

There are few reports on the role of HDAC9 in cartilage development, chondrocyte hypertrophy and OA. Nkx3.2/Bapx1 is a crucial protein for maintaining chondrocyte viability. The HDAC9-PIASy-RNF4 axis could promote chondrocyte hypertrophy by regulating the sumoylation and ubiquitination of Nkx3.2/Bapx1, leading to its degradation through the proteasome (Choi et al., 2016).

### HDAC10

**FIGURE 2:**
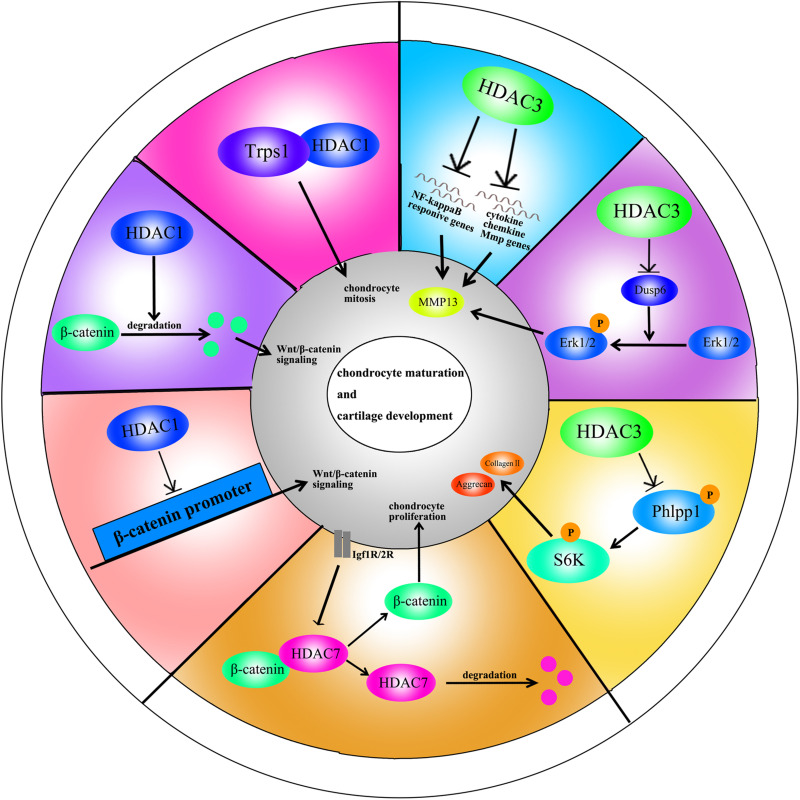
A schematic illustration of HDACs in chondrocyte maturation and cartilage development through different signaling pathways.

**FIGURE 3:**
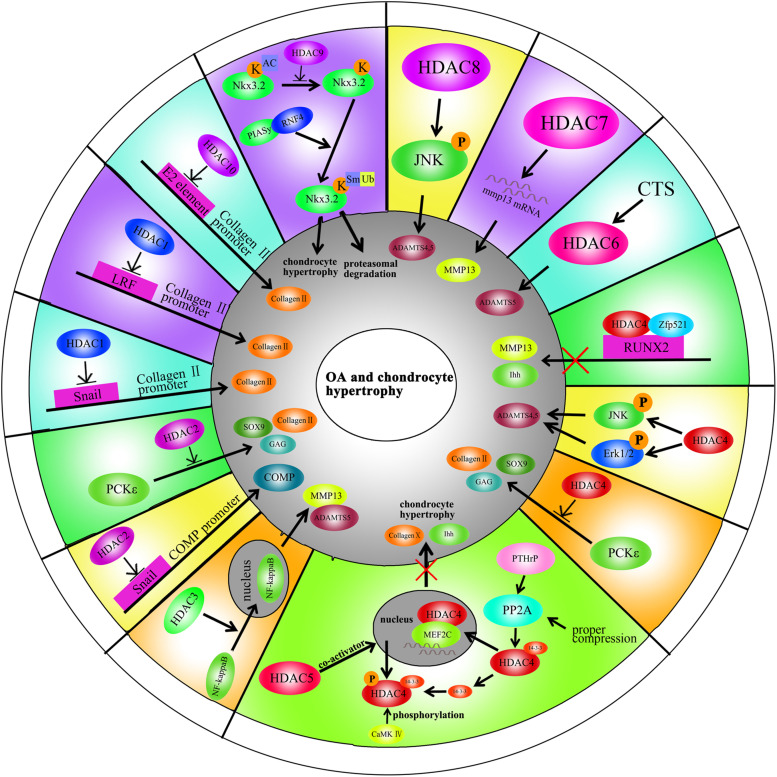
A schematic illustration of HDACs in OA and chondrocyte hypertrophy through different signaling pathways.

HDAC10 is involved in the regulation of collagen II expression through the epigenetic modification of enhancer elements in the collagen II gene. HDAC10 overexpression in Swarm rat chondrosarcoma (RCS) chondrocytes suppresses collagen II transcription through interaction with the E2 enhancer element in the collagen II gene, which locates 277 bp downstream of the transcription start site (Figures 2, 3; Nham et al., 2019).

## HDAC Inhibitors and OA Treatment

HDACi inhibit the enzymatic activity of HDACs and promote acetylation of proteins. HDACi can be divided into four groups, according to their structures: short-chain fatty acids, hydroxamic acids, cyclic peptides, and benzamides (Carpio and Westendorf, 2016). Currently, there have been more than 609 completed/ongoing HDACi-related human clinical trials, including trials for kidney disease (Chun, 2018), cardiovascular disease (Yoon and Eom, 2016), neuronal memory and regeneration (Ganai et al., 2016), myeloma and solid tumors (Hull et al., 2016; Cengiz Seval and Beksac, 2019). Although, there have been many studies on the effects of HDACi on OA, none of the HDACi identified have been approved as an OA treatment by the United States Food and Drug Administration (FDA).

The hydroxamic acids consist of trichostatin (TSA), vorinostat (SAHA), ricolinostat (ACY-1215) and givinostat (ITF2357). TSA is the most common broad spectrum HDACi. *In vitro*, TSA inhibits MMP1, MMP3, MMP13, and IL-1 in OA chondrocytes (Chen et al., 2010). One of the mechanisms of TSA treatment is that TSA abolishes the pro-inflammatory effect of Kruppel-like factor 4 (KLF4) (Fujikawa et al., 2017). Although TSA inhibits the inflammatory response in OA, it decreases collagen II mRNA levels in primary human chondrocyte stimulated with IL-1β or fibroblast growth factor-2 (FGF-2) (Wang et al., 2009). A Redox imbalance contributes to OA progression, TSA inhibits synthesis of NO and prostaglandin (PGE_2_), and the expression of inducible nitric oxide synthase (iNOS) and Cox-2 in chondrocytes stimulated with IL-1β (Chabane et al., 2008). Apoptosis is a crucial regulatory mechanism in OA. TSA suppresses apoptosis to protect chondrocytes (Song et al., 2015). In CTS-induced activation of the MAPK signaling pathway in chondrocytes, TSA downregulates MAPK and suppresses its downstream pro-inflammatory proteins (e.g., Runx2 and MMP13) at both the mRNA and protein levels (Saito et al., 2013). The protective effect of TSA is the same in leptin-stimulated human chondrocytes (Iliopoulos et al., 2007). *In vivo*, Cai et al. (2015) reported that TSA alleviates OA through the induction of Nrf2 and its downstream proteins. TSA also increases the Timp-1/MMP ratio in the OA model along with increased acetylation levels of H3 and H4. However, whether there is a relationship between histone acetylation and the Timp-1/MMP ratio needs additional study (Higashiyama et al., 2010). The protective effect of TSA was confirmed in an ACLT rabbit model through the inhibition of cathepsins (Chen et al., 2011). Furthermore, TSA also ameliorates OA by inhibiting synovial inflammation in an OA mouse model (Nasu et al., 2008).

Vorinostat (SAHA) is another HDACi composed of hydroxamic acids. It inhibits MMPs and iNOS by attenuating the NF-κB and MAPK pathways in human chondrocytes stimulated with IL-1β. However, vorinostat only inhibits p38 and Erk1/2 activation, not JNK activation (Zhong et al., 2013). Treatment with vorinostat also suppresses IL-6 expression in OA chondrocytes through the miR-9-MCPIP1 axis. Vorinostat promotes the recruitment of CEBPα to the promoter of MCPIIP1 to inhibit IL-1 synthesis (Makki and Haqqi, 2017). Furthermore, IL-6-induced MMP13 expression in the chondrocytes can be reversed by vorinostat, which promotes Col2a1 and ACAN expression in OA chondrocyte (Makki and Haqqi, 2016).

Ricolinostat (ACY-1215) is a selective HDAC6 inhibitor that has anti-inflammatory and chondroprotective properties. ACY-1215 inhibits MMP1 and MMP13 expression by down regulating NF-κB and STAT3 activity in primary human chondrocyte stimulated with IL-1β (Cheng et al., 2019).

Givinostat (ITF2357) is another anti-inflammatory compound that can inhibit MMPs expression in an experimental arthritis model (Joosten et al., 2011).

Butyrate acid is short-chain fatty acid that inhibits the expression of pro-inflammatory mediators, pro-inflammatory adipokines, and several inflammatory signaling pathways partly through G protein-coupled receptor (GPR)-43 (Pirozzi et al., 2018). Butyrate acid significantly abrogates IL-1β-induced MMPs at both the RNA and protein levels by inactivating NF-κB; however, butyrate acid inhibits Collagen II expression (Bo et al., 2018).

Valproic acid (VPA) is short-chain fatty acid, which inhibits HDAC1 activity and promotes the degradation of HDAC2 (Platta et al., 2008; Avery and Bumpus, 2014). VPA can prevent inflammatory damage to cartilage. Its protective role is achieved by the downregulation of microsomal prostaglandin E_2_ (Mpges-1) mediated by the induction of NAB1 in chondrocytes stimulated with IL-1β, which binds to the promoter of Mpges-1 (Zayed et al., 2011). VPA also represses cytokine-induced MMP1, MMP3, and MMP13 in human articular chondrocytes (HACs) (Culley et al., 2013).

Entinostat (MS-275) belongs to the benzamide class of compounds that selectively inhibits Class I HDACs (Lauffer et al., 2013). MS-275 inhibits MMP13 expression in OA (Queirolo et al., 2016), and prevents cartilage absorption (Culley et al., 2013). Furthermore, it suppresses CTS-induced expression of Runx2, ADAMTS5, and MMP3 at both the mRNA and protein levels in chondrocytes through inhibition of MAPK signaling pathway (Saito et al., 2013; Table 2).

**TABLE 2:** The classification of HDACi and effect of HDACi on OA.

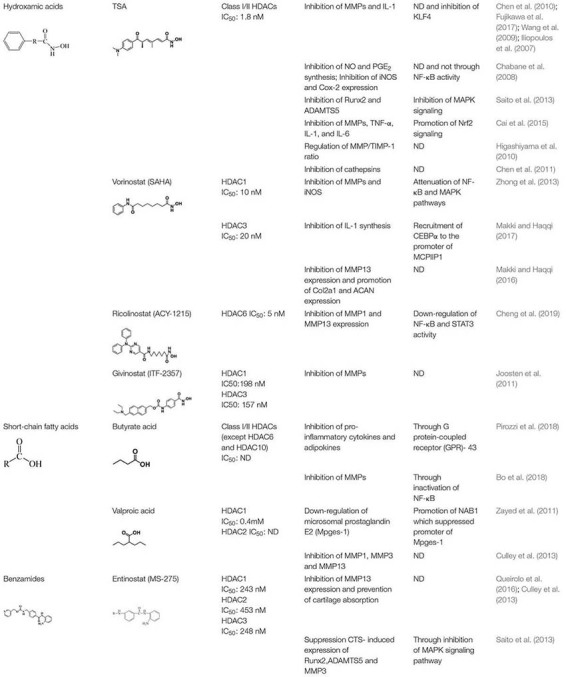

*ND: Not determined. IC_50_ and chemical structure were cited by https://www.medchemexpress.cn/.*

There are also some major obstacles to using HDACi to treat OA. HDACi affect many systems and organs through blood flow after administration. Inevitably, unnecessary side effects and toxicity occur, including secondary malignancies (Bhaskara et al., 2010; Mendivil et al., 2013). Most HDACi are broad-spectrum inhibitors. Thus, some beneficial HDACs may also be inhibited, leading to side effects and a low efficiency of HDACi therapy. Furthermore, the HDACi dosage should be determined for individual patients (Khabele, 2014). Too much or too little dosage drug will fail to achieve the expected therapeutic effects against OA.

## Conclusion

In recent years, HDACs have drawn more and more attention in the pathogenesis of OA. Previous studies demonstrated that HDACs not only regulate chondrocyte maturation and hypertrophy but also protect cartilage from damage. However, the mechanisms underlying the role of HDACs in OA are unclear and require additional investigation. Clarification of the roles of individual HDACs in cartilage will help define which HDAC(s) should be inhibited or activated for the treatment of OA. Moreover, a better understanding of the roles of individual HDACs in OA will reveal the major HDAC isoform(s) responsible for OA and allow the development of a selective HDACi to achieve a more precise and effective therapy for OA.

Based on this review, HDACi have protective roles in cartilage and enormous potential as new drugs to against OA. There are currently limitations to the use of HDACi as OA therapy. With non-selective HDACi, patients may suffer from severe side effect and toxicity. The dosage is an important consideration for the clinical use of HDACi. An unoptimized dosage might not achieve the predicted effect and could even cause harm. Therefore it’s essential to identify tissue-specific and HDAC-specific HDACi to avoid side effect and toxicity. In parallel, the development of specific HDACi will also help delineate the function of individual HDACs in OA. Finally, it’s necessary to build a drug evaluation system to guide dosage selection for individual patients to achieve the best therapeutic effect.

In conclusion, although our knowledge of OA continues to grow, understanding the underlying mechanisms involved in OA pathogenesis and identifying effective treatments will require further investigation. Based on current data, HDACs and HDACi hold promise for the management of OA.

## Author Contributions

HZ contributed to conception and design for the manuscript. HZ and LJ drafted the article. HZ, YY, and XZ contributed to critical revision of the article. YG and JL provided the technical support. LB approved the final version of the article. All authors contributed to the article and approved the submitted version.

## Conflict of Interest

The authors declare that the research was conducted in the absence of any commercial or financial relationships that could be construed as a potential conflict of interest.

## References

[B1] Angiolilli C. Baeten D. L. Radstake T. R. Reedquist K. A. (2017). The acetyl code in rheumatoid arthritis and other rheumatic diseases. *Epigenomics* 9 447–461. 10.2217/epi-2016-0136 28102705

[B2] Araki Y. Mimura T. (2017). Matrix metalloproteinase gene activation resulting from disordred epigenetic mechanisms in rheumatoid arthritis. *Int. J. Mol. Sci.* 18:905. 10.3390/ijms18050905 28441353 PMC5454818

[B3] Avery L. B. Bumpus N. N. (2014). Valproic acid is a novel activator of AMP-activated protein kinase and decreases liver mass, hepatic fat accumulation, and serum glucose in obese mice. *Mol. Pharmacol.* 85 1–10. 10.1124/mol.113.089755 24105977 PMC3868906

[B4] Bennell K. L. Egerton T. Martin J. Abbott J. H. Metcalf B. McManus F. (2014). Effect of physical therapy on pain and function in patients with hip osteoarthritis: a randomized clinical trial. *JAMA* 311 1987–1997. 10.1001/jama.2014.4591 24846036

[B5] Bennell K. L. Hunter D. J. Paterson K. L. (2017). Platelet-rich plasma for the management of hip and knee osteoarthritis. *Curr. Rheumatol. Rep.* 19:24. 10.1007/s11926-017-0652-x 28386761

[B6] Bhaskara S. Knutson S. K. Jiang G. Chandrasekharan M. B. Wilson A. J. Zheng S. (2010). Hdac3 is essential for the maintenance of chromatin structure and genome stability. *Cancer Cell* 18 436–447. 10.1016/j.ccr.2010.10.022 21075309 PMC3004468

[B7] Bo W. Zhou J. Wang K. (2018). Sodium butyrate abolishes the degradation of type II collagen in human chondrocytes. *Biomed. Pharmacother.* 102 1099–1104. 10.1016/j.biopha.2018.03.062 29710527

[B8] Bradley E. W. Carpio L. R. Olson E. N. Westendorf J. J. (2015). Histone deacetylase 7 (Hdac7) suppresses chondrocyte proliferation and beta-catenin activity during endochondral ossification. *J. Biol. Chem.* 290 118–126. 10.1074/jbc.M114.596247 25389289 PMC4281714

[B9] Bradley E. W. Carpio L. R. Westendorf J. J. (2013). Histone deacetylase 3 suppression increases PH domain and leucine-rich repeat phosphatase (Phlpp)1 expression in chondrocytes to suppress Akt signaling and matrix secretion. *J. Biol. Chem.* 288 9572–9582. 10.1074/jbc.M112.423723 23408427 PMC3617261

[B10] Brunmeir R. Lagger S. Seiser C. (2009). Histone deacetylase HDAC1/HDAC2-controlled embryonic development and cell differentiation. *Int. J. Dev. Biol.* 53 275–289. 10.1387/ijdb.082649rb 19412887

[B11] Cai D. Yin S. Yang J. Jiang Q. Cao W. (2015). Histone deacetylase inhibition activates Nrf2 and protects against osteoarthritis. *Arthritis Res. Ther.* 17:269. 10.1186/s13075-015-0774-3 26408027 PMC4583998

[B12] Cao K. Wei L. Zhang Z. Guo L. Zhang C. Li Y. (2014). Decreased histone deacetylase 4 is associated with human osteoarthritis cartilage degeneration by releasing histone deacetylase 4 inhibition of runt-related transcription factor-2 and increasing osteoarthritis-related genes: a novel mechanism of human osteoarthritis cartilage degeneration. *Arthritis Res. Ther.* 16:491. 10.1186/s13075-014-0491-3 25424126 PMC4265470

[B13] Carpio L. R. Bradley E. W. McGee-Lawrence M. E. Weivoda M. M. Poston D. D. Dudakovic A. (2016). Histone deacetylase 3 supports endochondral bone formation by controlling cytokine signaling and matrix remodeling. *Sci. Signal.* 9:ra79. 10.1126/scisignal.aaf3273 27507649 PMC5409103

[B14] Carpio L. R. Bradley E. W. Westendorf J. J. (2017). Histone deacetylase 3 suppresses Erk phosphorylation and matrix metalloproteinase (Mmp)-13 activity in chondrocytes. *Connect Tissue Res.* 58 27–36. 10.1080/03008207.2016.1236088 27662443 PMC5609188

[B15] Carpio L. R. Westendorf J. J. (2016). Histone deacetylases in cartilage homeostasis and osteoarthritis. *Curr. Rheumatol. Rep.* 18:52. 10.1007/s11926-016-0602-z 27402109

[B16] Cengiz Seval G. Beksac M. (2019). A comparative safety review of histone deacetylase inhibitors for the treatment of myeloma. *Expert Opin. Drug Saf.* 18 563–571. 10.1080/14740338.2019.1615051 31070945

[B17] Chabane N. Zayed N. Afif H. Mfuna-Endam L. Benderdour M. Boileau C. (2008). Histone deacetylase inhibitors suppress interleukin-1beta-induced nitric oxide and prostaglandin E2 production in human chondrocytes. *Osteoarthritis Cartilage* 16 1267–1274. 10.1016/j.joca.2008.03.009 18417374

[B18] Cheleschi S. De Palma A. Pecorelli A. Pascarelli N. A. Valacchi G. Belmonte G. (2017). Hydrostatic pressure regulates microRNA expression levels in osteoarthritic chondrocyte cultures via the Wnt/beta-catenin pathway. *Int. J. Mol. Sci.* 18:133. 10.3390/ijms18010133 28085114 PMC5297766

[B19] Chen C. Wei X. Wang S. Jiao Q. Zhang Y. Du G. (2016a). Compression regulates gene expression of chondrocytes through HDAC4 nuclear relocation via PP2A-dependent HDAC4 dephosphorylation. *Biochim. Biophys. Acta* 1863(7 Pt A), 1633–1642. 10.1016/j.bbamcr.2016.04.018 27106144 PMC4871159

[B20] Chen L. J. Wei S. Y. Chiu J. J. (2013). Mechanical regulation of epigenetics in vascular biology and pathobiology. *J. Cell. Mol. Med.* 17 437–448. 10.1111/jcmm.12031 23551392 PMC3822644

[B21] Chen W. Chen L. Zhang Z. Meng F. Huang G. Sheng P. (2016b). MicroRNA-455-3p modulates cartilage development and degeneration through modification of histone H3 acetylation. *Biochim. Biophys. Acta* 1863 2881–2891. 10.1016/j.bbamcr.2016.09.010 27638301

[B22] Chen W. Sheng P. Huang Z. Meng F. Kang Y. Huang G. (2016c). MicroRNA-381 regulates chondrocyte hypertrophy by inhibiting histone deacetylase 4 expression. *Int. J. Mol. Sci.* 17:1377. 10.3390/ijms17091377 27563877 PMC5037657

[B23] Chen W. P. Bao J. P. Hu P. F. Feng J. Wu L. D. (2010). Alleviation of osteoarthritis by Trichostatin A, a histone deacetylase inhibitor, in experimental osteoarthritis. *Mol. Biol. Rep.* 37 3967–3972. 10.1007/s11033-010-0055-9 20237852

[B24] Chen W. P. Bao J. P. Tang J. L. Hu P. F. Wu L. D. (2011). Trichostatin A inhibits expression of cathepsins in experimental osteoarthritis. *Rheumatol. Int.* 31 1325–1331. 10.1007/s00296-010-1481-7 20390279

[B25] Cheng C. Shan W. Huang W. Ding Z. Cui G. Liu F. (2019). ACY-1215 exhibits anti-inflammatory and chondroprotective effects in human osteoarthritis chondrocytes via inhibition of STAT3 and NF-kappaB signaling pathways. *Biomed. Pharmacother.* 109 2464–2471. 10.1016/j.biopha.2018.11.017 30551507

[B26] Cho H. J. Morey V. Kang J. Y. Kim K. W. Kim T. K. (2015). Prevalence and risk factors of spine, shoulder, hand, hip, and knee osteoarthritis in community-dwelling Koreans older than age 65 years. *Clin. Orthop. Relat. Res.* 473 3307–3314. 10.1007/s11999-015-4450-3 26162413 PMC4562942

[B27] Choi H. J. Kwon S. Kim D. W. (2016). A post-translational modification cascade employing HDAC9-PIASy-RNF4 axis regulates chondrocyte hypertrophy by modulating Nkx3.2 protein stability. *Cell. Signal.* 28 1336–1348. 10.1016/j.cellsig.2016.06.006 27312341

[B28] Chun P. (2018). Therapeutic effects of histone deacetylase inhibitors on kidney disease. *Arch. Pharm. Res.* 41 162–183. 10.1007/s12272-017-0998-7 29230688

[B29] Correa D. Hesse E. Seriwatanachai D. Kiviranta R. Saito H. Yamana K. (2010). Zfp521 is a target gene and key effector of parathyroid hormone-related peptide signaling in growth plate chondrocytes. *Dev. Cell* 19 533–546. 10.1016/j.devcel.2010.09.008 20951345 PMC2958174

[B30] Culley K. L. Wang H. Barter M. J. Davidson R. K. Swingler T. E. Destrument A. P. M. (2013). Class I histone deacetylase inhibition modulates metalloproteinase expression and blocks cytokine-induced cartilage degradation. *Arthritis Rheum.* 65 1822–1830. 10.1002/art.37965 23575963

[B31] de Ruijter A. J. van Gennip A. H. Caron H. N. Kemp S. van Kuilenburg A. B. (2003). Histone deacetylases (HDACs): characterization of the classical HDAC family. *Biochem. J.* 370(Pt 3), 737–749. 10.1042/BJ20021321 12429021 PMC1223209

[B32] Dvir-Ginzberg M. Mobasheri A. Kumar A. (2016). The role of sirtuins in cartilage homeostasis and osteoarthritis. *Curr. Rheumatol. Rep.* 18:43. 10.1007/s11926-016-0591-y 27289467

[B33] Fu S. Thompson, Ph D. C. Ali A. Wang, Ph D. W. (2019). Mechanical loading inhibits cartilage inflammatory signalling via an HDAC6 and IFT-dependent mechanism regulating primary cilia elongation. *Osteoarthritis Cartilage* 27 1064–1074. 10.1016/j.joca.2019.03.003 30922983 PMC6593179

[B34] Fujikawa J. Takeuchi Y. Kanazawa S. Nomir A. G. Kito A. Elkhashab E. (2017). Kruppel-like factor 4 regulates matrix metalloproteinase and aggrecanase gene expression in chondrocytes. *Cell Tissue Res.* 370 441–449. 10.1007/s00441-017-2674-0 28856432

[B35] Gallinari P. Di Marco S. Jones P. Pallaoro M. Steinkuhler C. (2007). HDACs, histone deacetylation and gene transcription: from molecular biology to cancer therapeutics. *Cell Res.* 17 195–211. 10.1038/sj.cr.7310149 17325692

[B36] Ganai S. A. Ramadoss M. Mahadevan V. (2016). Histone Deacetylase (HDAC) Inhibitors - emerging roles in neuronal memory, learning, synaptic plasticity and neural regeneration. *Curr. Neuropharmacol.* 14 55–71. 10.2174/1570159x13666151021111609 26487502 PMC4787286

[B37] Guan Y. Chen Q. Yang X. Haines P. Pei M. Terek R. (2012). Subcellular relocation of histone deacetylase 4 regulates growth plate chondrocyte differentiation through Ca2+/calmodulin-dependent kinase IV. *Am. J. Physiol. Cell Physiol.* 303 C33–C40. 10.1152/ajpcell.00348.2011 22442139 PMC3404523

[B38] Guan Y. J. Li J. Yang X. Du S. Ding J. Gao Y. (2018). Evidence that miR-146a attenuates aging- and trauma-induced osteoarthritis by inhibiting Notch1, IL-6, and IL-1 mediated catabolism. *Aging Cell* 17:e12752. 10.1111/acel.12752 29575548 PMC5946074

[B39] Hesham H. M. Lasheen D. S. Abouzid K. A. M. (2018). Chimeric HDAC inhibitors: comprehensive review on the HDAC-based strategies developed to combat cancer. *Med. Res. Rev.* 38 2058–2109. 10.1002/med.21505 29733427

[B40] Higashiyama R. Miyaki S. Yamashita S. Yoshitaka T. Lindman G. Ito Y. (2010). Correlation between MMP-13 and HDAC7 expression in human knee osteoarthritis. *Mod. Rheumatol.* 20 11–17. 10.1007/s10165-009-0224-7 19784544 PMC2818344

[B41] Hong S. Derfoul A. Pereira-Mouries L. Hall D. J. (2009). A novel domain in histone deacetylase 1 and 2 mediates repression of cartilage-specific genes in human chondrocytes. *FASEB J.* 23 3539–3552. 10.1096/fj.09-133215 19561124 PMC2747680

[B42] Huang X. Xu J. Huang M. Li J. Dai L. Dai K. (2014). Histone deacetylase1 promotes TGF-beta1-mediated early chondrogenesis through down-regulating canonical Wnt signaling. *Biochem. Biophys. Res. Commun.* 453 810–816. 10.1016/j.bbrc.2014.10.021 25445594

[B43] Hull E. E. Montgomery M. R. Leyva K. J. (2016). HDAC inhibitors as epigenetic regulators of the immune system: impacts on cancer therapy and inflammatory diseases. *Biomed. Res. Int.* 2016:8797206. 10.1155/2016/8797206 27556043 PMC4983322

[B44] Iliopoulos D. Malizos K. N. Tsezou A. (2007). Epigenetic regulation of leptin affects MMP-13 expression in osteoarthritic chondrocytes: possible molecular target for osteoarthritis therapeutic intervention. *Ann. Rheum. Dis.* 66 1616–1621. 10.1136/ard.2007.069377 17502362 PMC2095321

[B45] Jenei-Lanzl Z. Meurer A. Zaucke F. (2019). Interleukin-1beta signaling in osteoarthritis - chondrocytes in focus. *Cell. Signal.* 53 212–223. 10.1016/j.cellsig.2018.10.005 30312659

[B46] Jeong Y. Du R. Zhu X. Yin S. Wang J. Cui H. (2014). Histone deacetylase isoforms regulate innate immune responses by deacetylating mitogen-activated protein kinase phosphatase-1. *J. Leukoc. Biol.* 95 651–659. 10.1189/jlb.1013565 24374966

[B47] Johnson V. L. Hunter D. J. (2014). The epidemiology of osteoarthritis. *Best Pract. Res. Clin. Rheumatol.* 28 5–15. 10.1016/j.berh.2014.01.004 24792942

[B48] Joosten L. A. Leoni F. Meghji S. Mascagni P. (2011). Inhibition of HDAC activity by ITF2357 ameliorates joint inflammation and prevents cartilage and bone destruction in experimental arthritis. *Mol. Med.* 17 391–396. 10.2119/molmed.2011.00058 21327299 PMC3105133

[B49] Khabele D. (2014). The therapeutic potential of class I selective histone deacetylase inhibitors in ovarian cancer. *Front. Oncol.* 4:111. 10.3389/fonc.2014.00111 24904826 PMC4033132

[B50] Khan N. M. Haqqi T. M. (2018). Epigenetics in osteoarthritis: potential of HDAC inhibitors as therapeutics. *Pharmacol. Res.* 128 73–79. 10.1016/j.phrs.2017.08.007 28827187 PMC5803377

[B51] Kozhemyakina E. Cohen T. Yao T. P. Lassar A. B. (2009). Parathyroid hormone-related peptide represses chondrocyte hypertrophy through a protein phosphatase 2A/histone deacetylase 4/MEF2 pathway. *Mol. Cell. Biol.* 29 5751–5762. 10.1128/MCB.00415-09 19704004 PMC2772746

[B52] Kroesen M. Gielen P. Brok I. C. Armandari I. Hoogerbrugge P. M. Adema G. J. (2014). HDAC inhibitors and immunotherapy; a double edged sword? *Oncotarget* 5 6558–6572. 10.18632/oncotarget.2289 25115382 PMC4196144

[B53] Kuyinu E. L. Narayanan G. Nair L. S. Laurencin C. T. (2016). Animal models of osteoarthritis: classification, update, and measurement of outcomes. *J. Orthop. Surg. Res.* 11:19. 10.1186/s13018-016-0346-5 26837951 PMC4738796

[B54] Lauffer B. E. Mintzer R. Fong R. Mukund S. Tam C. Zilberleyb I. (2013). Histone deacetylase (HDAC) inhibitor kinetic rate constants correlate with cellular histone acetylation but not transcription and cell viability. *J. Biol. Chem.* 288 26926–26943. 10.1074/jbc.M113.490706 23897821 PMC3772242

[B55] Leus N. G. Zwinderman M. R. Dekker F. J. (2016). Histone deacetylase 3 (HDAC 3) as emerging drug target in NF-kappaB-mediated inflammation. *Curr. Opin. Chem. Biol.* 33 160–168. 10.1016/j.cbpa.2016.06.019 27371876 PMC5019345

[B56] Litwic A. Edwards M. H. Dennison E. M. Cooper C. (2013). Epidemiology and burden of osteoarthritis. *Br. Med. Bull.* 105 185–199. 10.1093/bmb/lds038 23337796 PMC3690438

[B57] Liu C. J. Prazak L. Fajardo M. Yu S. Tyagi N. Di Cesare P. E. (2004). Leukemia/lymphoma-related factor, a POZ domain-containing transcriptional repressor, interacts with histone deacetylase-1 and inhibits cartilage oligomeric matrix protein gene expression and chondrogenesis. *J. Biol. Chem.* 279 47081–47091. 10.1074/jbc.M405288200 15337766

[B58] Liu Q. Wang S. Lin J. Zhang Y. (2018). The burden for knee osteoarthritis among Chinese elderly: estimates from a nationally representative study. *Osteoarthritis Cartilage.* 26 1636–1642. 10.1016/j.joca.2018.07.019 30130589

[B59] Lyu X. Hu M. Peng J. Zhang X. Sanders Y. Y. (2019). HDAC inhibitors as antifibrotic drugs in cardiac and pulmonary fibrosis. *Ther. Adv. Chronic Dis.* 10:2040622319862697. 10.1177/2040622319862697 31367296 PMC6643173

[B60] Makki M. S. Haqqi T. M. (2016). Histone deacetylase inhibitor vorinostat (SAHA) suppresses IL-1beta-induced matrix metallopeptidase-13 expression by inhibiting IL-6 in osteoarthritis chondrocyte. *Am. J. Pathol.* 186 2701–2708. 10.1016/j.ajpath.2016.06.010 27555113 PMC5222971

[B61] Makki M. S. Haqqi T. M. (2017). Histone deacetylase inhibitor vorinostat (SAHA, MK0683) perturb miR-9-MCPIP1 axis to block IL-1beta-induced IL-6 expression in human OA chondrocytes. *Connect. Tissue Res.* 58 64–75. 10.1080/03008207.2016.1211113 27404795 PMC5233650

[B62] Malemud C. J. (2017). Negative regulators of JAK/STAT signaling in rheumatoid arthritis and osteoarthritis. *Int. J. Mol. Sci.* 18:484. 10.3390/ijms18030484 28245561 PMC5372500

[B63] Mao G. Hu S. Zhang Z. Wu P. Zhao X. Lin R. (2018). Exosomal miR-95-5p regulates chondrogenesis and cartilage degradation via histone deacetylase 2/8. *J. Cell. Mol. Med.* 22 5354–5366. 10.1111/jcmm.13808 30063117 PMC6201229

[B64] Mao G. Zhang Z. Huang Z. Chen W. Huang G. Meng F. (2017). MicroRNA-92a-3p regulates the expression of cartilage-specific genes by directly targeting histone deacetylase 2 in chondrogenesis and degradation. *Osteoarthritis Cartilage* 25 521–532. 10.1016/j.joca.2016.11.006 27884646

[B65] Mendivil A. A. Micha J. P. Brown J. V. III Rettenmaier M. A. Abaid L. N. Lopez K. L. (2013). Increased incidence of severe gastrointestinal events with first-line paclitaxel, carboplatin, and vorinostat chemotherapy for advanced-stage epithelial ovarian, primary peritoneal, and fallopian tube cancer. *Int. J. Gynecol. Cancer* 23 533–539. 10.1097/IGC.0b013e31828566f1 23385285

[B66] Meng F. Li Z. Zhang Z. Yang Z. Kang Y. Zhao X. (2018). MicroRNA-193b-3p regulates chondrogenesis and chondrocyte metabolism by targeting HDAC3. *Theranostics* 8 2862–2883. 10.7150/thno.23547 29774080 PMC5957014

[B67] Moon S. M. Lee S. A. Han S. H. Park B. R. Choi M. S. Kim J. S. (2018). Aqueous extract of Codium fragile alleviates osteoarthritis through the MAPK/NF-kappaB pathways in IL-1beta-induced rat primary chondrocytes and a rat osteoarthritis model. *Biomed. Pharmacother.* 97 264–270. 10.1016/j.biopha.2017.10.130 29091874

[B68] Nasu Y. Nishida K. Miyazawa S. Komiyama T. Kadota Y. Abe N. (2008). Trichostatin A, a histone deacetylase inhibitor, suppresses synovial inflammation and subsequent cartilage destruction in a collagen antibody-induced arthritis mouse model. *Osteoarthritis Cartilage* 16 723–732. 10.1016/j.joca.2007.10.014 18226559

[B69] Neogi T. (2013). The epidemiology and impact of pain in osteoarthritis. *Osteoarthritis Cartilage* 21 1145–1153. 10.1016/j.joca.2013.03.018 23973124 PMC3753584

[B70] Nham G. T. H. Zhang X. Asou Y. Shinomura T. (2019). Expression of type II collagen and aggrecan genes is regulated through distinct epigenetic modifications of their multiple enhancer elements. *Gene* 704 134–141. 10.1016/j.gene.2019.04.034 30981839

[B71] Ooi J. Y. Tuano N. K. Rafehi H. Gao X. M. Ziemann M. Du X. J. (2015). HDAC inhibition attenuates cardiac hypertrophy by acetylation and deacetylation of target genes. *Epigenetics* 10 418–430. 10.1080/15592294.2015.1024406 25941940 PMC4622459

[B72] Pirozzi C. Francisco V. Guida F. D. Gomez R. Lago F. Pino J. (2018). Butyrate modulates inflammation in chondrocytes via GPR43 receptor. *Cell. Physiol. Biochem.* 51 228–243. 10.1159/000495203 30448827

[B73] Platta C. S. Greenblatt D. Y. Kunnimalaiyaan M. Chen H. (2008). Valproic acid induces Notch1 signaling in small cell lung cancer cells. *J. Surg. Res.* 148 31–37. 10.1016/j.jss.2008.03.008 18570928 PMC2900385

[B74] Queirolo V. Galli D. Masselli E. Borzi R. M. Martini S. Vitale F. (2016). PKCepsilon is a regulator of hypertrophic differentiation of chondrocytes in osteoarthritis. *Osteoarthritis Cartilage* 24 1451–1460. 10.1016/j.joca.2016.04.003 27072078

[B75] Rigoglou S. Papavassiliou A. G. (2013). The NF-kappaB signalling pathway in osteoarthritis. *Int. J. Biochem. Cell Biol.* 45 2580–2584. 10.1016/j.biocel.2013.08.018 24004831

[B76] Saito T. Nishida K. Furumatsu T. Yoshida A. Ozawa M. Ozaki T. (2013). Histone deacetylase inhibitors suppress mechanical stress-induced expression of RUNX-2 and ADAMTS-5 through the inhibition of the MAPK signaling pathway in cultured human chondrocytes. *Osteoarthritis Cartilage* 21 165–174. 10.1016/j.joca.2012.09.003 23017871

[B77] Sarzi-Puttini P. Cimmino M. A. Scarpa R. Caporali R. Parazzini F. Zaninelli A. (2005). Osteoarthritis: an overview of the disease and its treatment strategies. *Semin. Arthritis Rheum.* 35(1 Suppl. 1), 1–10. 10.1016/j.semarthrit.2005.01.013 16084227

[B78] Shen J. Chen D. (2014). Recent progress in osteoarthritis research. *J. Am. Acad. Orthop. Surg.* 22 467–468. 10.5435/JAAOS-22-07-467 24966254 PMC4124725

[B79] Si H. B. Zeng Y. Liu S. Y. Zhou Z. K. Chen Y. N. Cheng J. Q. (2017). Intra-articular injection of microRNA-140 (miRNA-140) alleviates osteoarthritis (OA) progression by modulating extracellular matrix (ECM) homeostasis in rats. *Osteoarthritis Cartilage* 25 1698–1707. 10.1016/j.joca.2017.06.002 28647469

[B80] Sondag G. R. Haqqi T. M. (2016). The role of MicroRNAs and their targets in osteoarthritis. *Curr. Rheumatol. Rep.* 18:56. 10.1007/s11926-016-0604-x 27402113 PMC5294969

[B81] Song J. Jin E. H. Kim D. Kim K. Y. Chun C. H. Jin E. J. (2015). MicroRNA-222 regulates MMP-13 via targeting HDAC-4 during osteoarthritis pathogenesis. *BBA Clin.* 3 79–89. 10.1016/j.bbacli.2014.11.009 26673737 PMC4661531

[B82] Spange S. Wagner T. Heinzel T. Kramer O. H. (2009). Acetylation of non-histone proteins modulates cellular signalling at multiple levels. *Int. J. Biochem. Cell Biol.* 41 185–198. 10.1016/j.biocel.2008.08.027 18804549

[B83] Thomas A. C. Hubbard-Turner T. Wikstrom E. A. Palmieri-Smith R. M. (2017). Epidemiology of posttraumatic osteoarthritis. *J. Athl. Train.* 52 491–496. 10.4085/1062-6050-51.5.08 27145096 PMC5488839

[B84] Thompson C. L. Chapple J. P. Knight M. M. (2014). Primary cilia disassembly down-regulates mechanosensitive hedgehog signalling: a feedback mechanism controlling ADAMTS-5 expression in chondrocytes. *Osteoarthritis Cartilage* 22 490–498. 10.1016/j.joca.2013.12.016 24457103 PMC3988976

[B85] Toh W. S. Lai R. C. Hui J. H. P. Lim S. K. (2017). MSC exosome as a cell-free MSC therapy for cartilage regeneration: implications for osteoarthritis treatment. *Semin. Cell Dev. Biol.* 67 56–64. 10.1016/j.semcdb.2016.11.008 27871993

[B86] Wang H. Zhang H. Sun Q. Yang J. Zeng C. Ding C. (2018a). Chondrocyte mTORC1 activation stimulates miR-483-5p via HDAC4 in osteoarthritis progression. *J. Cell. Physiol.* 234 2730–2740. 10.1002/jcp.27088 30145794

[B87] Wang J. H. Shih K. S. Wu Y. W. Wang A. W. Yang C. R. (2013). Histone deacetylase inhibitors increase microRNA-146a expression and enhance negative regulation of interleukin-1beta signaling in osteoarthritis fibroblast-like synoviocytes. *Osteoarthritis Cartilage* 21 1987–1996. 10.1016/j.joca.2013.09.008 24107356

[B88] Wang P. Mao Z. Pan Q. Lu R. Huang X. Shang X. (2018b). Histone deacetylase-4 and histone deacetylase-8 regulate interleukin-1beta-induced cartilage catabolic degradation through MAPK/JNK and ERK pathways. *Int. J. Mol. Med.* 41 2117–2127. 10.3892/ijmm.2018.3410 29393346 PMC5810207

[B89] Wang X. Song Y. Jacobi J. L. Tuan R. S. (2009). Inhibition of histone deacetylases antagonized FGF2 and IL-1beta effects on MMP expression in human articular chondrocytes. *Growth Factors* 27 40–49. 10.1080/08977190802625179 19107653 PMC3612426

[B90] Wuelling M. Pasdziernik M. Moll C. N. Thiesen A. M. Schneider S. Johannes C. (2013). The multi zinc-finger protein Trps1 acts as a regulator of histone deacetylation during mitosis. *Cell Cycle* 12 2219–2232. 10.4161/cc.25267 23892436 PMC3755072

[B91] Yang C. R. Shih K. S. Liou J. P. Wu Y. W. Hsieh I. N. Lee H. Y. (2014). Denbinobin upregulates miR-146a expression and attenuates IL-1beta-induced upregulation of ICAM-1 and VCAM-1 expressions in osteoarthritis fibroblast-like synoviocytes. *J. Mol. Med.* 92 1147–1158. 10.1007/s00109-014-1192-8 25052989

[B92] Yang X. Guan Y. Tian S. Wang Y. Sun K. Chen Q. (2016). Mechanical and IL-1beta responsive miR-365 contributes to osteoarthritis development by targeting histone deacetylase 4. *Int. J. Mol. Sci.* 17:436. 10.3390/ijms17040436 27023516 PMC4848892

[B93] Yoon S. Eom G. H. (2016). HDAC and HDAC inhibitor: from cancer to cardiovascular diseases. *Chonnam Med. J.* 52 1–11. 10.4068/cmj.2016.52.1.1 26865995 PMC4742605

[B94] Zayed N. El Mansouri F. E. Chabane N. Kapoor M. Martel-Pelletier J. Benderdour M. (2011). Valproic acid suppresses interleukin-1ss-induced microsomal prostaglandin E2 synthase-1 expression in chondrocytes through upregulation of NAB1. *J. Rheumatol.* 38 492–502. 10.3899/jrheum.100907 21239760

[B95] Zhang C. Zhang Z. Chang Z. Mao G. Hu S. Zeng A. (2019a). miR-193b-5p regulates chondrocytes metabolism by directly targeting histone deacetylase 7 in interleukin-1beta-induced osteoarthritis. *J. Cell. Biochem.* 120 12775–12784. 10.1002/jcb.28545 30854734

[B96] Zhang H. Ji L. Yang Y. Wei Y. Zhang X. Gang Y. (2019b). The therapeutic effects of treadmill exercise on osteoarthritis in rats by inhibiting the HDAC3/NF-KappaB pathway *in vivo* and *in vitro*. *Front. Physiol.* 10:1060. 10.3389/fphys.2019.01060 31481898 PMC6710443

[B97] Zheng G. Zhan Y. Tang Q. Chen T. Zheng F. Wang H. (2018). Monascin inhibits IL-1beta induced catabolism in mouse chondrocytes and ameliorates murine osteoarthritis. *Food Funct.* 9 1454–1464. 10.1039/c7fo01892d 29473075

[B98] Zhong H. M. Ding Q. H. Chen W. P. Luo R. B. (2013). Vorinostat, a HDAC inhibitor, showed anti-osteoarthritic activities through inhibition of iNOS and MMP expression, p38 and ERK phosphorylation and blocking NF-kappaB nuclear translocation. *Int. Immunopharmacol.* 17 329–335. 10.1016/j.intimp.2013.06.027 23856614

